# Role of Calcium in an Experimental Breast Cancer Model Induced by Radiation and Estrogen

**DOI:** 10.3390/biomedicines12112432

**Published:** 2024-10-23

**Authors:** Gloria M. Calaf, Luis N. Ardiles, Leodan A. Crispin

**Affiliations:** Instituto de Alta Investigación, Universidad de Tarapacá, Arica 1000000, Chile; lardilesa@academicos.uta.cl (L.N.A.); kcrispi@gestion.uta.cl (L.A.C.)

**Keywords:** S100 proteins, Ca^2+^-dependent signaling, biomarkers, estrogen, breast cancer, radiation

## Abstract

**Background**: Breast cancer, a global health challenge, significantly impacts women worldwide, causing morbidity, disability, and mortality. **Objectives:** To analyze the role of genes encoding S100 calcium-binding proteins and their relationship with radiation as possible markers in breast carcinogenesis. **Methods:** The normal MCF-10F cell line was used to study the role of ionizing radiation and estrogen to induce distinct stages of malignancy giving rise to an in vitro experimental breast cancer model. **Results:** Analysis of an Affymetrix system revealed that the gene expression levels of the S100 calcium-binding protein P (*S100P*), the S100 calcium-binding protein A14 (S100A14), and the S100 calcium-binding protein A2 (*S100A2*) were greater in the Tumor2 than the non-tumorigenic Alpha3 or the tumorigenic Alpha5 cell lines; however, the S100 calcium-binding protein A8 (*S100A8*) and the S100 calcium-binding protein A9 (*S100A9*) expression levels were higher in A5 than T2 and A3 cell lines. A significant positive association was found between the estrogen receptor alpha gene (*ESR1*) and *S100A14* in Basal and Her2 patients. The association between *ESR1* and *S100A8* and *S100A9* expression levels was positive in Basal patients but negative in Her2, Luminal A, and Luminal B. *S100P* and *S100A14* expression levels were higher in tumor tissues than in normal ones. The estrogen receptor status was positive in patients with high levels of the *S10014* gene, but negative in *S100A2*, *S100A8*, and *S100A9* expression levels. **Conclusion:** Cell dependence needs to be considered while designing new breast cancer treatments since gene signatures might vary depending on the type of tumor.

## 1. Introduction

Breast cancer remains a significant global health challenge, exerting substantial morbidity, disability, and mortality among women worldwide [1]. The staggering statistics of approximately 2.3 million new cases and 685,000 deaths in 2020 underscore the urgency for comprehensive research into its molecular underpinnings [2,3]. At early stages, breast cancer or just spread to the axillary lymph nodes is still curable [4]. However, with advancements in endocrine therapy, treatment in the late stages when the cancer has spread to distant locations is still a challenge.

The S100 genes play an important role in tumorigenesis by regulating cell proliferation, invasion, metastasis, cell survival, or cell death; it includes at least 13 members located as a cluster on chromosome 1q21 [5]. The S100 family is a group of proteins widely studied due to its ability to modulate cellular processes in response to changes in calcium concentrations. Such interactions modulate calcium-binding protein expression and play a role in cancer among others [6]. Among them, the S100 calcium-binding proteins (S100) belong to a broad family of cytosolic proteins that bind to calcium and are associated with tumor progression and inflammation [7]. Calcium molecules are important ions that regulate multiple cellular functions from intracellular proliferation to muscle contraction. Calcium-binding proteins (CBPs) play a crucial role in mediating the effects of calcium, enabling its transport across cell membranes and decoding signals that are vital for cellular homeostasis. Within this group of proteins, the S100 family has been widely studied due to its ability to modulate cellular processes in response to changes in calcium concentrations. Several genes encode the S100 protein family, affecting crucial cellular processes and cancer progression, the S100 protein family plays an important role in carcinogenesis. The S100 proteins interact with multiple targets during several physiological activities, such as Ca^2+^ homeostasis, proliferation, differentiation, apoptosis, inflammation, and cell migration [8,9]; thus, almost all cellular functions rely on Ca^2+^, and high Ca^2+^ concentrations can cause cell death.

Authors [10] have outlined the connection between S100 genes and calcium relative to their diseases, hypothesizing that Ca^2+^ is important as it regulates various biological activities. Ca^2+^-binding sites regulate their function in a Ca^2+^-dependent manner, or specialized Ca^2+^ sensing proteins. Such proteins may regulate effector protein activity by Ca^2+^-dependent association or through post-translation modifications. Both modifications are displayed by enzymes that are regulated in a Ca^2+^-dependent manner either because they have a Ca^2+^-binding motif or are associated with Ca^2+^-binding proteins. Also, these authors found the relationship between the genes that encoded the S100 and calcium itself and their pathologies. The S100 proteins are localized in the cytoplasm and nucleus of a wide range of cells and regulate several cellular processes such as cell cycle progression and differentiation [11].

It has been reported that normal human breast epithelial cells have a limited ability to divide in culture conditions, and such cultured cells are considered mortal and undergo a progressive cessation of in vitro growth, becoming senescent, and then dying [12,13,14,15,16]. The spontaneously immortalized human breast epithelial cell line MCF-10F was isolated and characterized by ultrastructural and immunocytochemical analysis [13,17]. The mortal parental cells and the cultures of primary human breast epithelial cells expressed low levels of *S100P*, regardless of the calcium concentration in the medium. That is, the *S100P* gene has been reported to be overexpressed in immortal cells compared to mortal ones [18]. Then, the immortalization of S130 to MCF-10F cells was associated with the acquisition of independence from calcium concentration in the medium, the chromosomal translocation [13,17], mutation of p53 [19], and stabilization of telomere length [20], among others.

The EF-hand domains are the most prevalent Ca^2+^-binding motifs in proteins. This family of proteins performs several tasks, including signal transduction between compartments, nucleus-based gene expression, and cytoplasmic Ca^2+^ buffering. The Ca^2+^-binding motif, known as the EF-hand is composed of a Ca^2+^-coordinated loop surrounded by two nearly perpendicular α-helices [21]. Ca^2+^ binds to EF-hand domains with varying affinities, which explains several biological roles; these proteins perform in a wide range of [Ca^2+^] [22]. High affinity Ca^2+^-binding proteins function as Ca^2+^-buffer proteins, adjusting the duration and form of Ca^2+^ signals to support the preservation of Ca^2+^ homeostasis.

Multiple Ca^2+^-sensing elements decode the calcium signal, which is necessary for the selective control of particular targets. Specialized motifs on proteins with prior biochemical characterizations allow the detection of Ca^2+^. Human mutations and illnesses associated with several Ca^2+^-sensing proteins have been related to the Ca^2+^-sensing mechanisms, found inside and outside organelles [23]. The S100 proteins form a growing subfamily of proteins related by Ca^2+^-binding motifs to the EF-hand Ca^2+^-binding protein superfamily [24].

The S100A family members regulate multiple biological functions related to cancer progression and metastasis; however, the prognostic of such a family has not been systematically investigated in cancer [25]. Authors have found that the S100 gene family, which comprises over 20 members, including *S100A1*, *S100A2*, *S100A8*, and *S100A9* encoded low molecular weight calcium-binding proteins with important physiological and pathological roles in keratinization [26]; however, there has also been evidence of a correlation between breast cancer and *S100A2*, *S100A4*, *S100A6*, *S100A7*, *S100A8*, *S100A9*, and *S100A11* [27].

One of the members of the S100 family of proteins, containing 2 EF-hand calcium-binding motifs [28], is the S100 calcium-binding protein P gene (*S100P*). On the other hand, the S100 calcium-binding protein A14 gene (*S100A14*), which encodes a member of the S100 protein family, contains an EF-hand motif that binds calcium and is in a cluster of the S100 genes on chromosome 1. The *S100A14* has been shown to regulate cell cycle progression, cell proliferation, migration, and invasion, playing an important role in metastasis in cervical cancer [29].

Another member of the S100 family of proteins containing 2 EF-hand calcium-binding motifs is the S100 calcium-binging protein A2 gene (*S100A2*) [30], which seems to have a tumor suppressor function, and its chromosomal rearrangements and altered expression are implicated in breast cancer [30]. Other members of the S100 family of proteins containing 2 EF-hand calcium-binding motifs [7] are the S100 calcium-binding proteins A8 and A9 genes (*S100A8* and *S100A9*, respectively) which function as inhibitors of casein kinase [7,31].

The genes *S100P*, *S100A14*, *S100A2*, *S100A8*, and *S100A9* were analyzed in the MCF-10F (Ct), estrogen (E), Alpha3 (A3), Alpha5 (A5), and Tumor2 (T2) cell lines. Such cell lines come from an ionizing radiation and estrogen experimental breast cancer model established in 2000 by Calaf and Hei [32]. The model was used to determine whether radiation in the presence of estrogen induced neoplastic transformation by using a normal immortalized human breast cell line as the MCF-10F. Such a cell line was exposed to low doses of high LET alpha particles (150 keV/micron) since radiation, as an environmental carcinogen, has been considered a major etiological factor [33]. Thus, this study aimed to examine the genes mentioned above, which encode the S100 calcium-binding proteins, and their relationship with radiation to determine their potential relevance as markers in breast cancer progression.

## 2. Materials and Methods

### 2.1. Cell Lines

**Figure 1:**
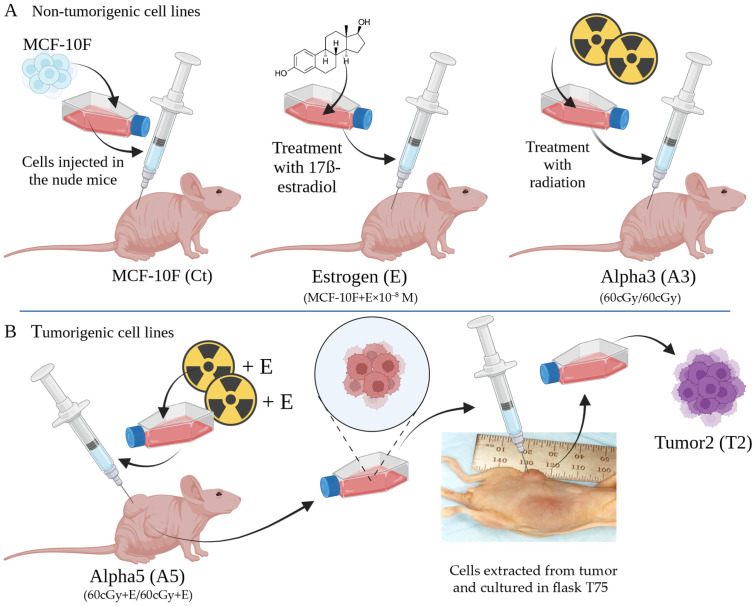
An experimental breast cancer model, a laboratory-based model induced by estrogen and radiation, yielded various experimental cell lines. These include (**A**) a non-cancerous cell line like the MCF-10F (Ct), that was not exposed to radiation; the estrogen (E) cell line, consistently treated with 17β-estradiol at a concentration of 10^−8^ mol/L; and the malignant cell line known as Alpha3 (A3), produced by irradiating MCF-10F cells with two 60/60 cGy α particle doses. (**B**) The cancerous cell lines such as Alpha5 (A5), which are MCF-10F cells that were exposed to two 60/60 cGy α particle doses in conjunction with estrogen, and the Tumor2 (T2) cell line, originated from mammary tumors appearing in nude mice after being injected with the A5 cell line. This image was created with Biorender (https://www.biorender.com/), accessed on 19 June 2024. Ct: control.

The immortalized human breast epithelial cell line MCF-10F (ATCC) was developed by Calaf and Hei in 2000 [32]. Such a cell line was exposed to low levels of high-linear-energy-transfer (LET) α particle radiation (150 keV/m and then, it was kept in culture for up to 10 months, in the presence of 17β-estradiol. After exposure, the MCF-10F cell line underwent several transformation stages, resulting in the various cell lines used in this study. These include (i) the MCF-10F (Control); (ii) the estrogen (E), MCF-10F consistently exposed to 10^−8^ mol/L 17β-estradiol; (iii) the non-tumorigenic but transformed Alpha3 (A3), MCF-10F subjected to two 60/60 cGy doses; (iv) the malignant and tumorigenic Alpha5 (A5), MCF-10F treated with two 60/60 cGy α particles in the presence of 17β-estradiol; and (v) the Tumor2 (T2) cell lines, a tumorigenic cell line derived from an A5 xenograft injected into nude mice [32]. A summary of these cell lines is provided in Figure 1. The cell lines were cultured with DMEM/F-12 (1:1) media that was enriched with the following antibiotics: 2.5 g/mL amphotericin B, 100 g/mL streptomycin, and 100 U/mL penicillin (all sourced from Life Technologies, Grand Island, NY, USA). Additionally, 0.02 g/mL of epidermal growth factor (from Collaborative Research, Bedford, MA, USA), 0.5 g/mL of hydrocortisone (from Sigma, St. Louis, MO, USA), and 10 g/mL and 5% horse serum (from Biofluids, Rockville, MD, USA) were used [13,32,34,35,36].

### 2.2. Irradiation

MCF-10F cells, which were undergoing rapid expansion, were plated in stainless steel rings of 60 mm diameter, each having a mylar bottom of 6 µm at the Radiological Research Facilities of Columbia University (also known as the Nevis Center). This was done three days before radiation and at a rate of 3 × 10^5^ cells per ring. The cells were subjected to gradient doses of ^4^He ions, with an energy of 150 KeV/µm, these ions were sped up to 4 MeV using the van de Graaff accelerator. Calaf and Hei carried out this procedure in 2000 [37]. Then the MCF-10F cells were exposed to one or two doses of ^4^He ions, each dose being 30, 60, or 100 cGy, and there was a gap of 12- to 14 weeks between the treatments. The irradiated cultures were promptly subcultured to assess the growth kinetics and to continue observing them for any changes in phenotype. Samples were frozen in anticipation of future use. The surviving cells were then passaged for more radiation therapy and samples were taken to look for various phenotypes that had changed. Cells were then cultured with or without estrogen after that.

### 2.3. Preparation of Fluorescence-Labeled Probes for the Analysis of Cell Lines

The Poly(A) mRNA was extracted from normal, radiation-exposed, and estrogen-exposed breast cell lines using the QIA-Direct-mRNA Isolation kit from Qiagen, based in Valencia, CA, USA. A fluorescently-tagged cDNA was synthesized from one microgram of these poly(A)mRNAs using oligo dT-initiated polymerization and the Superscript II reverse transcriptase kit from Life Technologies, located in Grand Island, NY, USA. This process was carried out in the presence of either Cy3- or Cy5-tagged dCTP, following the standard protocol as described earlier [38]. The Cy3 and Cy5-tagged probes were then combined, attached to the microarray on glass coverslips, and incubated for 16 h at a temperature of 65 °C. They were then rinsed and readied for scrutiny.

### 2.4. Examining Microarray Gene Expression with the Affymetrix HG-U133A Plus 2.0 GeneChip

The cell lines from this experimental model were developed by Calaf and Hei in 2000 [32]. Then in 2013, such cell lines, including the MCF-10F, the Estrogen, the Alpha3, the Alpha5, and the Tumor2 were subjected to a gene expression analysis using the Affymetrix U133A oligonucleotide microarray (Affymetrix, Santa Clara, CA, USA), which boasts a remarkable 14,500 genes. The arrays for gene expression were analyzed [38] with the Affymetrix GeneChip Operating Software (GCOS) v1.0 ST, the Genes@Work software (v 1.0) interface, and the SPLASH (structural pattern localization analysis by sequential histograms) discovery algorithm, all while maintaining a false discovery rate of 0.05 [39].

### 2.5. Bioinformatic and Statistical Analysis

A web-based program called Tumor Immune Estimation Resource v2.0 (TIMER2.0) (http://timer.cistrome.org/), accessed on 6 July 2024, reference number [40], was used to systematically examine immune infiltrates across different forms of cancer. Its three main components are the Immune Association, Cancer Exploration, and Immune Estimation. The Gene_Corr, Gen_DE, and Gene_Outcome modules are included in the Cancer Exploration section. A list of genes associated with various subtypes of breast cancer and a target gene were correlated, according to the Gene_Cor module. The statistical analysis of such correlations was carried out using Spearman’s test. This module created a heatmap chart that showed the links between the target gene and the group of genes being examined, and extensive information about any connection in the chart can be easily visualized by selecting the relevant entry. When the ‘purity adjusted’ option is chosen, selecting the figures in the chart will produce two scatter diagrams indicating (i) the correlation of the specified gene expression with tumor purity (the proportion of cancer cells in a sample) and (ii) the correlation of the gene expression with the other selected genes.

The Gene_DE module exhibited the variances in gene expression between healthy tissue and cancerous growths. This module utilized box plots to demonstrate the spread of gene expression levels, with the Wilcoxon test being used to determine the statistical significance. The results were denoted with a series of stars (* *p* < 0.05, ** *p* < 0.01, *** *p* < 0.001). The Gene_Outcome module evaluated the outcome pertinence of gene expression that was altered by the stage clinical factor, leveraging the Cox proportional hazard model. The ER status of the genes that were part of this study was provided by the University of California, Santa Cruz through the UCSC Xena Functional Genomics Explorer (http://xena.ucsc.edu/), reference number [41]. The one-way ANOVA test was used to calculate the statistical significance. *p* < 0.05 was considered statistically significant.

## 3. Results

### 3.1. Differential Gene Expression of Genes That Code for the S100 Calcium-Binding Proteins

**Figure 2:**
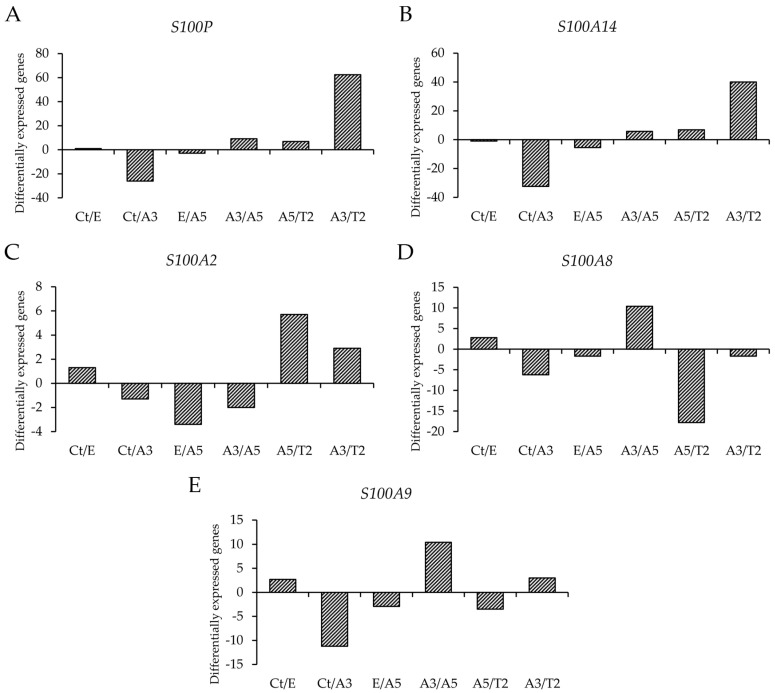
Affymetrix array (U133A) was used to profile genes that were differentially expressed such as (**A**) the S100 calcium-binding protein P (*S100P*), (**B**) the S100 calcium-binding protein A14 (*S100A14*), (**C**) the S100 calcium-binding protein A2 (*S100A2*), (**D**) the S100 calcium-binding protein A8 (*S100A8*), and (**E**) the S100 calcium-binding protein A9 (*S100A9*) in the cell lines as follow: Control/Estrogen (Ct/E), Control/Alpha3 (Ct/A3), Estrogen/Alpha5 (E/A5), Alpha3/Alpha5 (A3/A5), Alpha5/Tumor2 (A5/T2), and Alpha3/Tumor2 (A3/T2). The graphs include data taken from a cluster dendrogram collection of gene expression in our laboratory for this article.

In this study, the cell lines utilized were derived from the experimental model induced by radiation and estrogen, a model established by Calaf and Hei in 2000 [32]. In the year 2013, a pairwise comparative study of various cell lines [38] was conducted using the Affymetrix HG U-133A oligonucleotide microarray. The cell line pairs under study were MCF-10F/estrogen (Ct/E); MCF-10F/Alpha3 (Ct/A3); estrogen/Alpha5 (E/A5); Alpha3/Alpha5 (A3/A5); Alpha5/Tumor2 (A5/T2), and Alpha3/Tumor2 (A3/T2) as seen in Figure 2. These pairs were chosen based on the following criteria: (i) The pair MCF-10F/E was used to analyze the estrogen effect; (ii) MCF-10F/Alpha3, to analyze radiation alone; (iii) E/Alpha5, to assess the radiation effect when combined with estrogen; (iv) Alpha3/Alpha5, to analyze radiation plus estrogen combined versus radiation alone; (v) Alpha 3/Tumor2, to evaluate the impact of radiation alone and the microenvironment; and (vi) to assess the relationship between estrogen and ionizing-radiation combined and the environment in the athymic animal, Alpha5/Tumor2.

The analysis of Figure 2 shows the differential expression of the genes under investigation, which revealed that the T2 cell line expressed higher expression of the *S100P* gene than the A3 cell line. The T2 and Ct had higher *S100A14* expression than the A3. The *S100A2* expression was also higher in the T2 cell line in comparison with A3 and A5. On the other hand, the A5 cell line showed high levels of *S100A8* expression compared to T2 and A3; and A5 and Ct showed higher levels of *S100A9* expression levels than the A3 cell line.

### 3.2. Clinical Significance and Gene Expression in Different Breast Cancer Subtypes

#### 3.2.1. Comparison of the Genes in This Study with the Estrogen Receptor Alpha Gene

**Figure 3:**
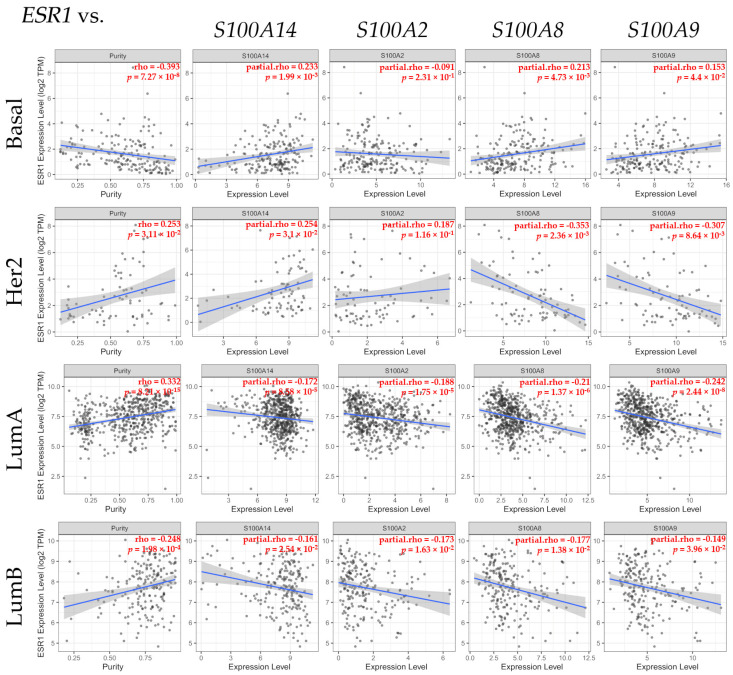
The scatter plots show the correlation values, with linear regression lines, of *ESR1* and the expression levels of the S100 calcium-binding protein A14 gene (*S100A14*), S100 calcium-binding protein A2 gene (*S100A2*), S100 calcium-binding protein A8 gene (*S100A8*), and S100 calcium-binding protein A9 gene (*S100A9*) in invasive breast carcinoma and the purity adjustment (Purity column). The blue lines in each plot indicate the linear regression fit, which depicts the pattern or relationship between the expression level of *ESR1* and the related gene. The gray-shaded area around each blue regression line represents the confidence range. The correlation analysis for each box is shown in red in the upper right corner. The statistical significance was determined using TIMER2.0. (Spearman, *p* < 0.05), reference number [40], accessed 14 March 2024.

The Gen Corr module of TIMER2.0 was used to identify the association between the estrogen receptor alpha gene (*ESR1*) and *S100P*, *S100A14*, *S100A2*, *S100A8*, and *S100A9* gene expression levels (Figure 3).

Scatter plot graphs show the association between the *ESR1* gene expression and the genes under study as seen in Figure 3. There was no significant association between *ESR1* and *S100P* expression. However, a significant (*p* < 0.05) positive correlation between *ESR1* and *S100A14* gene expression was found in Basal and Her2 cancer patients, but such a correlation was significantly (*p* < 0.05) negative in Luminal A and Luminal B patients. A significant (*p* < 0.05) negative association between *ESR1* and *S100A2* gene expression was found in Luminal A and Luminal B patients. Furthermore, a significant (*p* < 0.05) positive correlation between *ESR1* and *S100A8* and *S100A9* gene expression levels was observed in basal breast cancer patients; whereas such a correlation was significantly (*p* < 0.05) negative in the Her2, Luminal A, and Luminal B types of breast cancer patients.

#### 3.2.2. Gene Expression in Tumor Versus Normal Tissues

**Figure 4:**
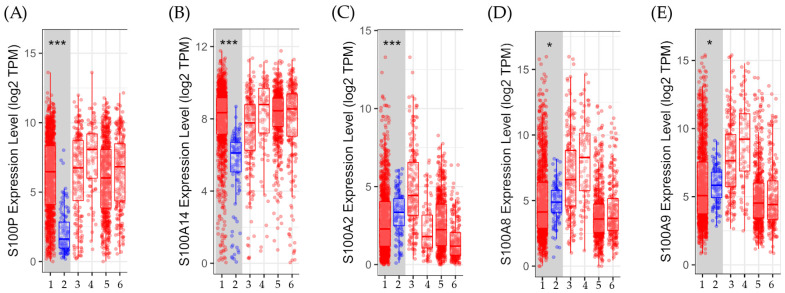
Determination of gene expression between tumor and normal tissues in different subtypes of breast cancer. The box plots show the distribution of gene expression levels of (**A**) the S100 calcium-binding protein P gene (*S100P*), (**B**) the calcium-binding protein A14 (*S100A14*), (**C**) the S100 calcium-binding protein A2 (*S100A2*), (**D**) the calcium-binding protein A8 (*S100A8*), and (**E**) the calcium-binding protein A9 (*S100A9*) in breast invasive carcinoma. TIMER2.0, reference number [40], accessed on 14 March 2024, established such levels using the Wilcoxon rank-sum test (*: *p* < 0.05, ***: *p* < 0.001). 1: Tumor: *n* = 1093, 2: Normal: *n* = 112, 3: Basal. Tumor: *n* = 190, 4: Her2. Tumor *n* = 82, 5: Luminal A. Tumor: *n* = 564, 6: Luminal B. Tumor: *n* = 217.

The Gen_DE module of TIMER2.0 was used to quantify the differential gene expression between normal and tumor tissues in breast cancer, as shown in Figure 4.

Gene expression comparisons between normal and tumor tissues across different subtypes are displayed in Figure 4. Results show that the expression levels of *S100P* and *S100A14* were significantly (*p* < 0.001) higher in the tumor tissue compared to the normal tissues. On the other hand, the levels of gene expression for *S100A2*, *S100A8*, and *S100A9* were significantly (either *p* < 0.001 or *p* < 0.05) greater in normal tissues than in malignancies.

#### 3.2.3. The Estrogen Receptor Status and Gene Expression

**Figure 5:**
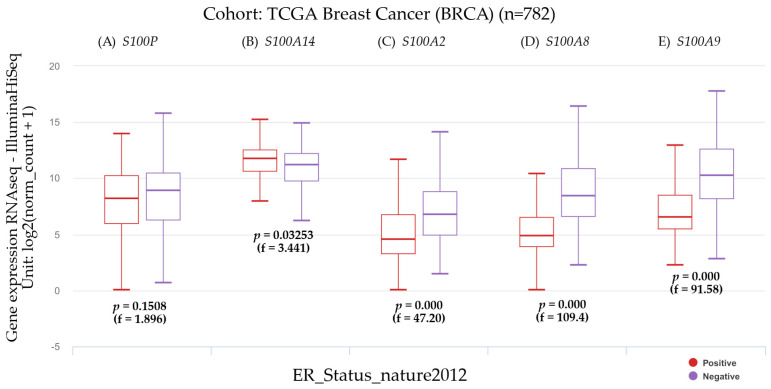
The state of the estrogen receptor and gene expression of (**A**) S100 calcium-binding protein P (*S100P*), (**B**) S100 calcium-binding protein A14 (*S100A14*), (**C**) S100 calcium-binding protein A2 (*S100A2*), (**D**) S100 calcium-binding protein A8 (*S100A8*), and (**E**) S100 calcium-binding protein A9 (*S100A9*) in breast invasive carcinoma stratified by Nature2012. Extracted from Xena functional genomics explorer (https://xena.ucsc.edu/), reference number [41], accessed on 14 March 2024.

Estrogen receptor status and the genes that encode for the S100 calcium-binding proteins are shown in Figure 5. Results were estimated by UCSC Xena tools.

Patients showing high *S100P* expression levels had no significant differences concerning the ER status as shown in Figure 5. However, patients with high *S100A14* gene expression showed a significant (*p* < 0.05) positive ER status. In contrast, the ER status in those patients having high *S100A2*, *S100A8*, and *S100A9* gene expression levels was significantly (*p* < 0.001) negative.

#### 3.2.4. Gene Expression and the Disease Stage Factor

The Cox proportional hazard model and TIMER2.0 were used to assess the survival difference of gene expression in breast cancer patients (*n* = 1100) adjusted by clinical stage factor (Table 1).

The clinical stages of patients with breast invasive cancer were taken into consideration when analyzing the gene expressions. The results showed that in Basal patients, the expression levels of *S100P*, *S100A14*, *S100A2*, *S100A8*, and *S100A9* were non-significant, but at stage 4, these expressions were significantly higher in Her2, Luminal A, and Luminal B patients (* *p* < 0.05, ** *p* < 0.01, or *** *p* < 0.001).

## 4. Discussion

The present study analyzed differential gene expression in the cell lines derived from an experimental breast cancer model, previously established, as well as the clinical parameters related to such genes in breast cancer patients. These studies are important since the evaluation and understanding of the roles of different genes can be useful as biomarkers in cancer development. This experimental breast cancer model originated in the year 2000 by using the MCF-10F cell line that was exposed to low doses of high LET α particle radiation in the presence of 17β-estradiol. After being exposed to either one or two doses of 60 cGy alpha particles, the MCF-10F cell line in the presence of estrogens underwent some stages of transformation before turning tumorigenic in nude mice [32]. 

The S100 proteins are also associated with inflammation and tumor progression, including breast cancer [7]. They also have a significant role in developing tumors, as demonstrated in vivo rat experiments [42]. Authors [28] have described that *S100P* encodes a calcium-binding protein expressed in different tumor tissues and functionally involved in the malignant phenomenon, which corroborates the present results.

The differential expression of genes revealed that the T2 cell line expressed higher expression of the *S100P* gene than the A3 cell line. The T2 cell line, which originated in the immunologically depressed mice, had higher *S100P* gene expression than the non-tumorigenic A3 cell line; such results corroborated the fact that the *S100P* gene expression was present in patients with metastasis making the probability of survival considerably lower in patients with invasive breast carcinoma as suggested by another study [43]. Furthermore, the correlation between gene expression and the disease stage of invasive carcinoma in different types of breast cancer indicated that the *S100P* gene expression showed no significance in Basal breast cancer patients but such expression was higher in Her2, Luminal A, and Luminal B subtypes at stage 4. There was no significance in the correlation between *ESR1* and *S100P* gene expression in any cancer patient subtypes, indicating no relationship with ERα, corroborated by the no significant difference between patients with high *S100P* gene expression levels and ER status. 

However, *S100P* gene expression was higher in tumor tissue than in the normal one, which is important in order to consider this gene an important biomarker for breast cancer.

Interestingly, these results are also supported by Peng et al. [44] where *S100P* was reported as a novel prognostic marker of metastatic breast cancer since it was found in high levels in the plasma of patients. Furthermore, Yang et al. [45] found an association between breast cancer and *S100P* methylation in peripheral blood by multicenter case-control studies. Thus, *S100P* can be considered an excellent marker for breast cancer at the level of biopsy, blood, and/or plasma.

A study [12] reported that *S100P* over-expression was an early event in the immortalization process of human mammary epithelial cells in vitro when comparing the immortal cell line MCF-10F to its mortal counterpart S130 or other primary cultures of human breast epithelial cells, where the clone was overexpressed. Furthermore, *S100P* was highly expressed above those found in the mortal S130 cells in all the immortal, chemically transformed breast epithelial cell lines [34], which included MCF-10F, BP1-E, and D3-1 and in three invasive ductal carcinomas and additional cell lines like T47D in comparison to the normal surrounding tissue. Then, such a study concluded that these immortalized and malignant cells had higher S100P levels than primary cultures of human breast epithelial cells. The S100P protein was thought to be one molecule implicated in particular cell cycle regulation pathways whose imbalance would allow cells to escape senesce and achieve an immortal cell state [46,47].

According to authors [42], overexpression of the S100P protein has been linked to both the in vitro immortalization of human breast epithelial cells and the in vivo early phases of breast cancer development. Using a monoclonal antibody against the same amino acid sequence of the cloned gene, the S100P protein was then localized by immunohistochemistry in ductal hyperplasia, in situ, and invasive ductal carcinoma, but not in the normal tissues. These studies led to the conclusion that S100P overexpression was a precursor that could be crucial for the in vivo growth of tumors and the in vitro immortalization of human breast epithelial cells [42]. Through bioinformatic analysis, it was observed that the S100P expression was increased in invasive ductal carcinomas when compared with the adjacent normal tissues [48,49].

Findings showed that the T2 cell line exhibited higher *S100A14* gene expression than the A3 cell line. Additionally, clinical data revealed a positive association between *ESR1* and *S100A14* gene expression in patients with Basal and Her2 breast cancer, but a negative correlation in patients with Luminal A and Luminal B breast cancer. Authors found that the S100A14 protein played a role in cell invasion by influencing the expression and functionality of matrix metalloproteinase (MMP)-2 [50]; as well as enhancing the motility of breast cancer cells by increasing the *S100A14* gene level [51]; furthermore, it enhanced the invasive activity of breast cancer cells through its interaction with cytoskeletal dynamics, suggesting its potential as a prognostic biomarker and a possible target for therapeutic interventions [52]. Others reported that the *S100A14* signaling promoted the metastasis of breast cancer [53] when serum levels were higher in patients with advanced breast cancer in comparison to those with localized one [54]; then, high levels of *S100A14* expression were linked to lower survival rates in breast cancer patients [55]. Authors identified the *S100A14* as an independent predictor of triple-negative breast cancer prognosis, a subgroup that generally has unfavorable outcomes and does not respond to targeted therapy, becoming a potential new treatment target for this cancer [56].

*S100A2* expression was higher in the T2 cell line compared to A3 and A5. Interestingly, *S100A2* expression was more commonly observed in breast cancer tissues compared to normal tissues, suggesting that this expression might serve as a cancer indicator in patients who demonstrated a substantial negative correlation between *ESR1* and *S100A2* expression in Luminal A and Luminal B breast cancer patients. Authors have demonstrated that S100A2 is one of several proteins that play a causal role in metastasis since they are characteristic of highly metastatic tumors [57]. However, in our bioinformatics analysis, normal tissues had higher levels of *S100A2* gene expression when compared to tumors, and other authors [58] confirmed S100A2 protein expression in normal human breast epithelium, but not in breast carcinoma cell lines. Others showed that the S100A2 was expressed in normal breast tissue but it was down-regulated during breast cancer progression [59]. The analysis also indicated that *S100A2* gene expression did not have significance in Basal breast cancer patients. However, it was higher in Her2, Luminal A, and Luminal B patients in stage 4 than in other clinical stages. 

The current study showed that *S100A8* expression was greater in the A5 cell line compared to the A3 and T2 and correlated in certain aspects with clinical subtypes in breast cancer patients [31]. However, clinical stages of patients with breast invasive carcinoma indicated gene expression was higher in Her2, Luminal A, and Luminal B patients in stage 4 but no significance at any stage in Basal breast cancer patients.

Other authors found increased *S100A8* gene and protein expression in breast cancer cells and the stroma of breast tumors [60]. The expression of the S100A8 protein was linked with a notably poorer prognosis in cancerous cells. The amplification of S100A8 did not seem connected with the expression of the S100A8 protein in breast cancer [61]. However, a tissue microarray analysis of human breast cancer showed a connection between the expression of the *S100A8* gene and unfavorable outcomes [62].

Results showed that *S100A9*, a major regulator of inflammation, had higher gene expression in the A5 than in the A3 cell line, indicating the role of estrogen in these processes since the difference between these two cell lines in the estrogen treatment along the process. The *S100A9* also plays a role in cancer progression and metastasis since it has been demonstrated to be expressed in the lungs [63]. Authors [64] reported *S100A9* in tumor-negative breast cancer that was highly lethal due to its aggressive clinical phenotype and the lack of validated therapeutic targets. According to the results, patients with stage 4 Her2, Luminal A, and Luminal B had greater levels of *S100A9* gene expression. Nonetheless, in patients with basal breast cancer, there was no significance.

Normal tissues had higher levels of *S100A8* and *S100A9* gene expression than malignancies did. It has also been shown that *S100A8*/*A9*, *S100A9*, and *S100A8* have the potential to be tumor diagnostic or prognostic biomarkers [65]. The heterodimer of the calcium-binding proteins S100A8 and S100A9, known as S100A8/A9, was first identified by the authors as an immunogenic protein that neutrophils produced and secreted [66]. In patients with basal carcinoma, the current study demonstrated a positive link between *ESR1* and the expression of the *S100A8* and *S100A9* genes, but a negative correlation with Her2, Luminal A, and Luminal B patients. Furthermore, *S100A8* and *S100A9* gene expression showed an ER-negative status in breast cancer patients. Studies have demonstrated increased *S100A8* and *S100A9* levels seen in patients with ER-negative breast cancer [31,67]. It was proposed that *S100A8* and *S100A9* expression represented a potential mechanism of the infiltrating myeloid cells having a clinical relevance, especially in tumor-negative breast cancer of basal-like subtypes [67,68]. 

The *S100A8* and *S100A9* members are among the S100 inflammatory proteins shown to modulate several breast cancer processes as progression and malignancy [69]. Both are among the most induced immune mediators involved in tumor stroma. Breast tumors with *ESR1* mutations displayed increased basal cytokeratins and immunological activation [70]. Cormier et al. [71] associated *S100A8* and *S100A9* members with breast cancer in a genome-wide transcriptome study and showed that both members could signal and regulate cancer cell behavior through the extracellular and intracellular-initiated cascades. Such authors elucidated the roles of intracellularly produced *S100A8* and *S100A9* on critical signaling pathways and biological mechanisms responsible for the malignancy of breast cancer. They found that adding S100A8 and S100A9 proteins to the MCF7 breast cancer cell line extracellularly promoted cell growth [71]. Growth inhibition resulted from the intracellular recombinant expression of *S100A8* and *S100A9*. Additionally, they demonstrated that *S100A8* and *S100A9* expressed intracellularly induced the expression of important markers including Zona occludens-1 (ZO-1) and Integrin alpha-5, which in turn encouraged an epithelial-like phenotype [71].

Furthermore, *S100A8* and *S100A9* altered the characteristics of cell adhesion and invasion by negatively regulating the pre-malignant Focal Adhesion Kinase-1 (FAK) signaling cascade activity. Significant variations were discovered between the effects of extracellular vs. intracellular initiated *S100A8* and *S100A9* signaling cascades on the development of mammary epithelial cells. Since the S100 protein has been linked to breast cancer invasiveness and metastasis, the *S100A8* and *S100A9* appeared to reduce breast cancer malignancy by increasing mesenchymal to epithelial transitioning [71].

**Figure 6:**
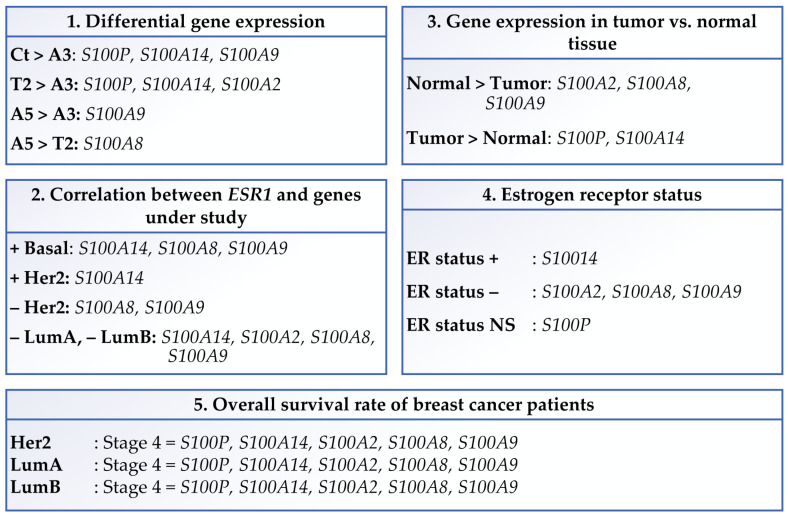
An overview of the key conclusions drawn from the Affymetrix array and their practical significance. (**1**) Differential gene expression was found in the control (Ct), estrogen (E), Alpha3 (A3), Alpha5 (A5), and tumor2 (T2) cell lines. (**2**) Association between *S100P*, *S100A14*, *S100A2*, *S100A8*, and *S100A9* expression levels and the expression of the *ESR1* gene in breast cancer subtypes. (**3**) Gene expression in tumor versus normal tissues. (**4**) Status of estrogen receptors. (**5**) Patients and breast cancer survival. Abbreviations, **+**: positive, **−**: negative, NS: non-significant, LumA: Luminal A, LumB: Luminal B.

Finally, Figure 6 presents a summary of the main findings of this study that shows the differential gene expression of *S100P*, *S100A14*, *S100A2*, *S100A8*, and *S100A9*, their correlation with *ESR1* gene expression, their expression in tumor versus normal tissues, the ER status, and the overall survival of patients.

## 5. Conclusions

Studies conducted in the last few years have demonstrated the critical role of the S100 proteins in a wide range of cellular functions and pathophysiological mechanisms. Results showed that ionizing radiation and estrogen affected the expression of those genes that encoded the S100 calcium-binding proteins such as *S100P*, *S100A14*, *S100A2*, *S100A8*, and *S100A9* in the immortalized breast cancer cell line MCF-10F. The clinical analysis indicated that among them, the *S100A14* gene could serve as an effective marker for cancer development at early stages for Lumina A patients and later stages for Her2 breast cancer patients. Several strategies have been developed to take advantage of the current understanding of the S100 proteins as useful partners in the context of cancer therapy. However, much more investigation is required to uncover and further optimize safe and effective S100 therapies as well as to universally establish S100 proteins as biomarkers.

## Figures and Tables

**Table 1:** Expression of genes and disease stage in different subtypes of breast cancer.

Cancer	*S100P*	*S100A14*	*S100A2*	*S100A8*	*S100A9*
All breast subtypes (*n* = 1100)	3, 4 ***	3, 4 ***	3, 4 ***	3, 4 ***	3, 4 ***
Basal (*n* = 191)	N.S.	N.S.	N.S.	N.S.	N.S.
Her2 (*n* = 82)	4 **	4 *	4 **	4 *	4 *
Luminal A (*n* = 568)	4 ***	4***	4 **	4 ***	4 ***
Luminal B (*n* = 219)	4 **	4 **	4 **	4 **	4 **

3, 4: breast cancer stages, N.S.: non-significant, *: *p* < 0.05, **: *p* < 0.01, ***: *p* < 0.001.

## Data Availability

Data concerning the clinical relevance presented in this study are openly available in TIMER2.0 (http://timer.cistrome.org), reference number [40] (accessed on 6 July 2024); UCSC Xena online exploration tools are freely available at http://xena.ucsc.edu/, reference number [41] (accessed on 20 June 2024). The data generated in the present study may be requested from the corresponding author.
